# Host Resistance and Temperature-Dependent Evolution of Aggressiveness in the Plant Pathogen *Zymoseptoria tritici*

**DOI:** 10.3389/fmicb.2017.01217

**Published:** 2017-06-28

**Authors:** Fengping Chen, Guo-Hua Duan, Dong-Liang Li, Jiasui Zhan

**Affiliations:** Fujian Key Laboratory of Plant Virology, Institute of Plant Virology, Fujian Agriculture and Forestry UniversityFuzhou, China

**Keywords:** *Septoria tritici*, evolution of plant pathogen, natural selection, host resistance, temperature-dependent, trade-offs

## Abstract

Understanding how habitat heterogeneity may affect the evolution of plant pathogens is essential to effectively predict new epidemiological landscapes and manage genetic diversity under changing global climatic conditions. In this study, we explore the effects of habitat heterogeneity, as determined by variation in host resistance and local temperature, on the evolution of *Zymoseptoria tritici* by comparing the aggressiveness development of five *Z. tritici* populations originated from different parts of the world on two wheat cultivars varying in resistance to the pathogen. Our results show that host resistance plays an important role in the evolution of *Z. tritici*. The pathogen was under weak, constraining selection on a host with quantitative resistance but under a stronger, directional selection on a susceptible host. This difference is consistent with theoretical expectations that suggest that quantitative resistance may slow down the evolution of pathogens and therefore be more durable. Our results also show that local temperature interacts with host resistance in influencing the evolution of the pathogen. When infecting a susceptible host, aggressiveness development of *Z. tritici* was negatively correlated to temperatures of the original collection sites, suggesting a trade-off between the pathogen’s abilities of adapting to higher temperature and causing disease and global warming may have a negative effect on the evolution of pathogens. The finding that no such relationship was detected when the pathogen infected the partially resistant cultivars indicates the evolution of pathogens in quantitatively resistant hosts is less influenced by environments than in susceptible hosts.

## Introduction

Plants and pathogens are engaged in a continuous co-evolutionary battle, with pathogens evolving new approaches to attack plants and plants responding through enhanced protection to prevent or mitigate damage (Zhan et al., 2014, 2015). As a consequence, pathogens serve as strong selective agents to regulate host density, diversity and defense mechanisms and plant hosts are among key drivers shaping the evolutionary landscapes of pathogens (Altizer et al., 2003; Garbelotto, 2012). Aggressiveness, defined as a quantitative component of pathogenicity, and measured by the amount of damage caused to plant hosts (Andrivon et al., 2007; Pariaud et al., 2009), is an important life-history trait resulting from the integrated effect of pathogen colonization, development and reproduction on hosts. It is an overall measurement of pathogen fitness composing of infection efficiency, latent period, sporulation rate, infectious period and lesion size (Pariaud et al., 2009) and plays an important role in host-pathogen co-evolution. Knowing evolutionary patterns and the causes of pathogen aggressiveness will provide useful insights into the dynamics of host-pathogen interactions and effective management of pathogen epidemiology and nature resources under changing climates globally (Pariaud et al., 2009; Zhan et al., 2015).

Many host genetic, physiological, biological and demographic characters can influence the evolutionary landscape of pathogen aggressiveness. Among them, host resistance, through its impact on key biological and ecological stochasticities of pathogens such as survival strategies (Carlsson-Graner and Thrall, 2015), reproductive modes (Zhan et al., 2007) and competitive abilities (Zhan and McDonald, 2013), is believed to be one of most important biotic factors shaping the population and evolutionary structure of pathogens (McDonald and Linde, 2002). Though fitness costs have been documented theoretically and experimentally (Dallas et al., 2016), resistance polymorphisms in host plants have been widely observed in natural ecosystems. In agro-ecosystems, resistance has been widely deployed as an artificial means to “compensate” for the host’s relative slowness to respond in the co-evolutionary “arms-race” with pathogens due to the host’s longer generation time and smaller population size compared to their pathogen adversaries (Greischar and Koskella, 2007; Vos et al., 2009).

Over co-evolutionary history, plants have evolved two major forms of host resistance to increase their fitness when encountering pathogens (Poland et al., 2009; Lo Iacono et al., 2012). Qualitative host resistance, expressed as compatible or incompatible relationships between hosts and pathogens following the gene-for-gene model, was first documented in the flax-rust pathosystem (Flor, 1956) and its roles in disease epidemiology and the evolution of pathogens have since attracted considerable theoretical and experimental attention. In contrast, empirical studies linking the impact of quantitative resistance to pathogen population genetic dynamics and evolution have largely been neglected. Quantitative resistance is usually seen as continuous variation in the mitigation of disease development and is thought to result from interactions among multiple genes each contributing minor but additive effects to host defense (Poland et al., 2009; Niks et al., 2015). Theoretically, quantitative host resistance is assumed to select for higher pathogen aggressiveness because it decreases the pathogen’s basic reproduction rate (Gandon and Michalakis, 2000). However, evidence from empirical results is inconsistent. Some data do indicate natural selection for a generalist host genotype with pathogen populations originating from quantitatively resistant hosts displaying significantly higher aggressiveness than those from susceptible hosts (Yang et al., 2013; Delmotte et al., 2014). However, other studies lean more toward the development of host specialists with pathogen populations adapting to dominant or parental host genotypes regardless of the types of host resistance they originated from (Ahmed et al., 1995, 1996; Andrivon et al., 2007). Quantitative host resistance is also thought to be more durable due to its reduced intensity of selection against pathogens (Wolfe and McDermott, 1994).

In addition to host, temperature is one of the most important environmental parameters having critical impacts on nearly all aspects of biological (Nadeem et al., 2014), ecological (Loehle et al., 2016) and biochemical processes (Park et al., 2011). In host-pathogen interactions, temperature can regulate the occurrence, development and severity of disease epidemics in the short-term through its impacts on pathogen metabolic rates and the expression of virulence factors, etc. (Smirnova et al., 2001; Nicholls et al., 2011). In the long-term, it can also exert influence on the evolutionary trajectory of pathogens such as their adaptation to thermal conditions (Zhan and McDonald, 2011; Yang et al., 2016), emergence of novel physiological races (Wu et al., 2016), sensitivity to agrochemicals (Qin et al., 2016) and variation in aggressiveness (Schade et al., 2014). Temperature-mediated pathogen aggressiveness has been documented in some plant pathogens. For example, novel wheat stripe rust isolates collected after 2000 displayed higher aggressiveness especially at higher temperatures than those collected before 2000, possibly associated with the increase in global temperatures detected in recent decades (Milus et al., 2009). However, it is not clear whether this is a general or specific pattern, or how temperature may interact with other factors such as host resistance in determining the evolution of pathogen aggressiveness. Such knowledge is urgently required particularly in light of the high probability that average air temperatures will continue to increase in coming decades, accompanied by a greater frequency of extreme temperature events (Savary et al., 2012; Gautam et al., 2013).

In this study, we used the *Zymoseptoria tritici* (anamorph *Septoria tritici*)-wheat system to test the hypotheses that plant resistance and local thermal conditions can affect the evolutionary trajectory of aggressiveness in plant pathogens. *Z. tritici* is among the most destructive pathogens of wheat, causing *S. tritici* leaf blotch. It is considered to be a rapidly evolving pathogen due to its high genetic variation generated by frequent sexual recombination (Chen and McDonald, 1996; Zhan et al., 2007), high gene flow (Zhan et al., 2003) and large effective population sizes (Zhan and McDonald, 2004). Under favorable conditions, the pathogen can cause yield losses of up to 40% (Eyal, 1981). Currently the disease is mainly controlled by the use of host resistance supplemented with fungicide application (Orton et al., 2011). Both quantitative and qualitative resistances have been identified in the wheat host (Risser et al., 2011; Kelm et al., 2012), but the majority of cultivars used commercially carry quantitative resistance.

The main objective of this study was to infer the role of genetic variation, host resistance and temperatures on the evolution of pathogens by comparing: (1) aggressiveness development of *Z. tritici* from geographic locations varying in thermal conditions: (2) the association between the genetic variation and mean of aggressiveness in *Z. tritici;* and (3) the amount and spatial distribution of genetic variation in neutral molecular markers and aggressiveness on two wheat cultivars differing in *Z. tritici* resistance.

## Materials and Methods

### Fungal Populations

Five *Z. tritici* populations including one each from Israel, Australia and Switzerland and two from Oregon, United States were used in this study. These populations originated from different locations in the world varying in climate and agricultural practices including the use of resistant genes. The Israeli population (ISR) was sampled from a wheat field located at Nahal Oz in 1992. The Australian population (AUS) was collected from a farm near Wagga Wagga in 2001 and the Swiss population (SWI) was sampled from a field located at Berga Irchel near Winterthur in 1999. The two United States populations (ORER and ORES) were collected on the same day in 1990 from a field planted with two different wheat cultivars at Corvallis, OR, United States. ORER was collected from the partially resistant cultivar Madsen while ORES was collected from the highly susceptible cultivar Stephens. All five populations have been characterized previously using restriction fragment length polymorphism (RFLP) markers and DNA fingerprints. A total of 151 genetically distinct isolates were selected from the five populations for aggressiveness assay. Each population was represented by 19–36 isolates each differing in their RFLP profiles (Linde et al., 2002; Zhan et al., 2003).

### Aggressiveness Tests

Aggressiveness of the pathogen, measured by the percentage of leaf area covered by lesion (PLACL), was tested on two Swiss wheat cultivars (Toronit and Greina) varying in level of resistance to *Z. tritici* (Zhan et al., 2005; Yang et al., 2013), Toronit was classified as moderately resistant to *Z. tritici*, while cultivar Greina was susceptible. Groups of ten seeds were sown separately in plastic pots filled with Ricoter garden soil (Ricoter Erdaufbereitung AG, Switzerland). After inoculation the pots were placed in a greenhouse at 60% RH and 20°C during the day, and 40% RH and 16°C at night.

For inoculation, *Z. tritici* isolates retrieved from long-term storage were placed on yeast maltose agar plates (YMA, 4 g/L yeast extract, 4 g/L malt extract, 4 g/L sucrose, 15 g/L agar) amended with 50 mg/L kanamycin and kept at 20°C for 1 week until blastospores formed. Blastospores were collected and transferred into sterile flasks containing 50 ml yeast sucrose broth (YSB, 10 g/L sucrose, 10 g/L yeast extract, 50 mg/L kanamycin). The inoculated flasks were maintained in an incubator at 20°C for 1 week before spores were harvested.

Spore suspensions amended with 0.04% Tween 20 (a non-toxic wetting agent) were adjusted to 5 × 10^6^ spores/mL on the day of inoculation using a haemocytometer. Inoculation was conducted appropriately 3 weeks after sowing when the seedlings were at growth stage 11 (Zadoks et al., 1974). Seedlings in each pot were thinned to the five most uniform and then inoculated by spraying with 10 ml of the spore suspension using a semi-automatic sprayer. Five pots (replicates) were inoculated for each cultivar-isolate combination. The inoculated pots were arranged according to Completely Randomized Design and placed at 100% RH and 20°C for 2 days in dark growth chambers before being returned to the original greenhouse. New leaves appearing after inoculation were removed at 3-day intervals.

Twenty-two days after inoculation, 1–2 inoculated top leaves were collected from each plant, and photographed with a digital camera. PLACLs were measured with the image analysis software Assess 2.0. All inoculations and digital images were made during a single day to minimize environmental variance among treatments. The detailed procedure for aggressiveness testing of these isolates has been described in the previous publications (Yang et al., 2013; Zhan et al., 2016).

### Data Analysis

Restriction fragment length polymorphism data for the fungal isolates were derived from previous publications (Zhan et al., 2003, 2005) but using only the isolates included in this study. Gene diversity (Nei, 1978) and genetic differentiation in the RFLP loci were estimated using Popgen 3.2 (Yeh et al., 1997). *G*_ST_ was calculated for each pair of populations as well as across all populations. Phenotypic variance of aggressiveness on each cultivar was partitioned into sources attributable to isolate (I, random effect) and population (P, random effect) using SAS GLM and VARCOMP programs (SAS Institute, 1990) according to the model:

$$Y_{rip}=M+I(P)+P+E_{rip}$$

where *Y*_rip_, *M*, *P*, *I*(*P*), *E*_rip_ is the mean PLACL of replicate *r* for isolate *i* in population *p*, the overall population mean, genetic variance among populations, genetic variance within populations, and the variance among replicates, respectively. In common garden experiments with asexually reproducing species, any variance among replicates can be attributed to environmental effects because individuals in different replicates have the same genotype (Zhan et al., 2005; Zhan and McDonald, 2011). Therefore, variance among replicates in this case is equivalent to the environmental variance of PLACL. Population differentiation in aggressiveness was estimated with following formula (Gonzalez-Martinez et al., 2002; Zhan et al., 2005; Zhan and McDonald, 2011):

$$Q_{ST}=\frac{\delta_{AP}^{2}}{\delta_{AP}^{2}+\delta_{WP}^{2}}$$

Where δ^2^_AP_ is the genetic variance in PLACL attributed to among population variation and δ^2^_WP_ is the genetic variance in PLACL attributed to within population variation.

Like *G*_ST_, *Q*_ST_ for PLACL was also calculated for all possible pairs of populations as well as across all populations. Heritability was estimated by dividing genetic variance within populations by total phenotypic variance (Falconer and Mackay, 1996). Statistical difference between overall *G*_ST_ in RFLP loci and overall *Q*_ST_ in aggressiveness was evaluated using the standard deviation of *Q*_ST_ constructed from 100 resampling of original data as described previously (Zhan and McDonald, 2011).

Monthly temperatures (mean, maximum and minimum) presented as an average over 10–15 years for each collection site were downloaded from World Climate^1^. For the ISR, temperature information was not available for Nahal Oz, Israel. In this case, the temperature data for Beer-Sheva, a location approximately 50 km east of Nahal Oz, was used (Zhan and McDonald, 2011). Variation in temperatures (mean, maximum and minimum) at each collection site was estimated based on the temperatures for each month.

Least significant difference was used to compare PLACL among populations sampled from different regions and hosts (Ott, 1992). Associations between pairwise differences in gene diversity and aggressiveness, between population differentiation for RFLP loci (*G*_ST_) and aggressiveness (*Q*_ST_), as well as between annual temperature and aggressiveness were evaluated using Pearson correlation (Lin, 1989).

## Results

### Variations in RFLP Markers and Aggressiveness of *Zymoseptoria tritici* Populations

**FIGURE 1:**
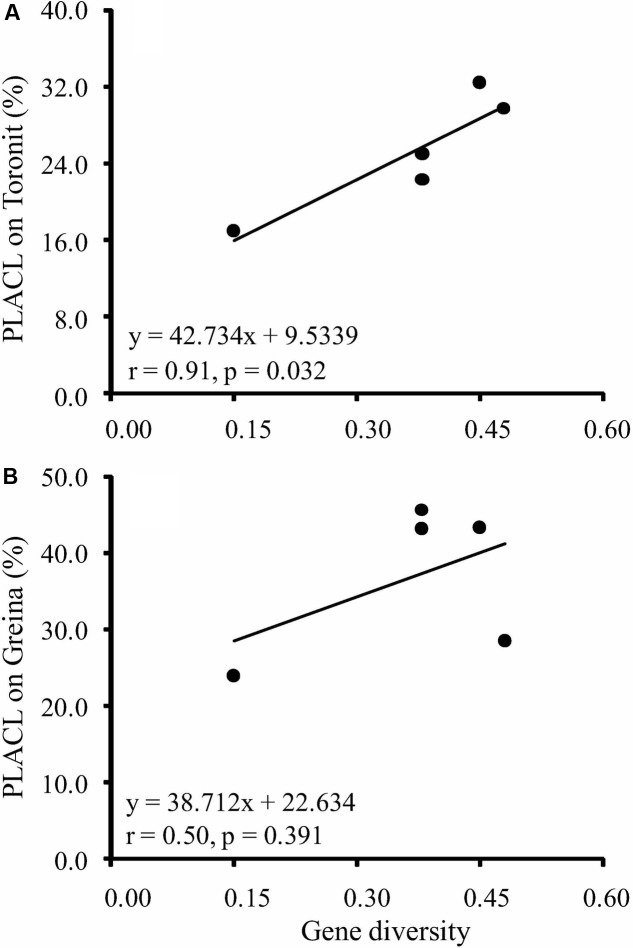
Correlation between gene diversity of RFLP markers and aggressiveness measured by the percentage of leaf area covered by lesion of five *Z. tritici* populations on two wheat cultivars varying in level of resistance. **(A)** Mean aggressiveness on a moderately resistant wheat cultivar Toronit; **(B)** mean aggressiveness on a susceptible wheat cultivar Greina.

Gene diversity in the five *Z. tritici* populations ranged from 0.15 to 0.48 (**Table 1**), where the AUS possessed the lowest and the ISR the highest diversity. The two populations ORER and ORES collected from the same location but different host cultivars in Oregon, United States had the same gene diversity. All isolates induced typical *S. tritici* leaf blotch symptoms on the two cultivars. At the isolate level, PLACL ranged from 1.0 to 90.3% with an average of 25.3% on the moderately resistant cultivar Toronit and from 2 to 90% with an average of 36.9% on the susceptible cultivar Greina. The average coefficient of PLACL variance among isolate replicates was 0.51, suggesting aggressiveness test is sensitive to environmental conditions and adequate replicates is required for a robust estimate of aggressiveness. The average PLACL for the five *Z. tritici* populations ranged from 16.9 to 32.4% and 23.9 to 45.6% on the Toronit and Greina cultivars, respectively. All populations showed higher PLACL on the susceptible cultivar Greina than on the resistant cultivar Toronit except the population from Israel which displayed similar PLACLs values (28.5 and 29.7 for Greina and Toronit, respectively). Heritability of aggressiveness ranged from 0.425 to 0.633 on Toronit, with an average of 0.543, and from 0.459 to 0.682 on Greina with an average of 0.574. Higher aggressiveness heritability on Greina was found for all *Z. tritici* populations with the exception of ORER (**Table 1**). Positive correlations between gene diversity and aggressiveness in the five *Z. tritici* populations were observed on both Toronit and Greina (**Figures 1A,B**) but only that involving aggressiveness on the resistant cultivar was significant (*r* = 0.91, *p* = 0.032).

**Table 1:** Genetic variation in restriction fragment length polymorphism (RFLP) markers and heritability of aggressiveness measured by the percentage of leaf area covered by lesion in the five *Z. tritici* populations on a moderately resistant wheat cultivar Toronit and a susceptible wheat cultivar Greina.

Population	Number of isolates	Gene diversity	PLACL (%)		Heritability of PLACL	
				
			Toronit	Greina	Toronit	Greina
AUS	31	0.15	16.9b^∗^	23.9a	0.425	0.459
ORER	36	0.38	25.0b	45.6a	0.633	0.562
ORES	19	0.38	22.3b	43.1a	0.565	0.652
SWI	32	0.45	32.4b	43.3a	0.630	0.682
ISR	33	0.48	29.7a	28.5a	0.462	0.517
Mean	30.2	0.37	25.3	36.9	0.543	0.574


*^∗^Values followed by different letters in the same row showing the significant difference analyzed by *t*-test at *p* = 0.05.*

### Population Genetic Differentiation in RFLP Loci and Aggressiveness in *Z. tritici*

**FIGURE 2:**
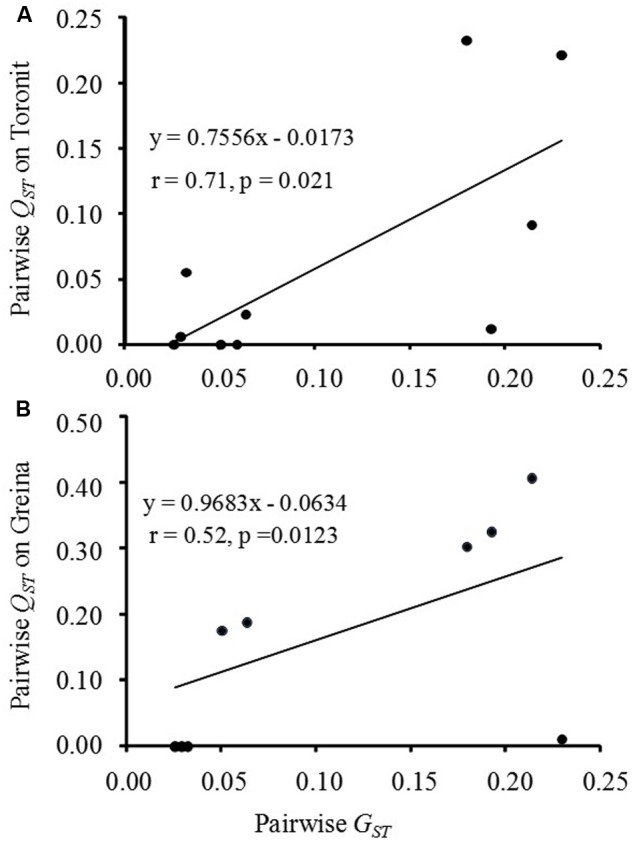
Pairwise comparisons between population differentiations for RFLP marker loci (*G*_ST_) and aggressiveness measured by the percentage of leaf area covered by lesion (*Q*_ST_) in the five *Z. tritici* populations on two cultivars. **(A)** Moderately resistant wheat cultivar Toronit; **(B)** susceptible wheat cultivar Greina.

Mean pairwise population differentiation (*G*_ST_) across the eight RFLP marker loci ranged from 0.03 to 0.23 with a grand mean of 0.11 (**Table 2**). The pairwise population differentiation (*Q*_ST_) in aggressiveness ranged from 0.00 to 0.23 in Toronit and from 0.00 to 0.41 Greina, with a mean of 0.06 and 0.17, respectively. Most pairwise *Q*_ST_ measures of aggressiveness in Toronit were smaller than *G*_ST_ while most *Q*_ST_ measures of aggressiveness in Greina were greater than *G*_ST_ (**Table 2**). The overall *G*_ST_ across the five populations was 0.114, which was significantly higher than overall *Q*_ST_ (0.059) in Toronit but significantly lower than overall *Q*_ST_ (0.173) in Greina. The correlation between *G*_ST_ and *Q*_ST_ was significantly positive in Toronit (*r* = 0.71, *p* = 0.021, **Figure 2A**) but not significant in Greina (*r* = 0.52, *p* = 0.123, **Figure 2B**).

**Table 2:** Pairwise comparisons between population differentiations for RFLP marker loci (*G*_ST_) and aggressiveness measured by the percentage of leaf area covered by lesion (*Q*_ST_) in the five *Z. tritici* populations on a moderately resistant wheat cultivar Toronit and a susceptible wheat cultivar Greina.

	ORER	ORES	ISR	AUS	SWI
ORER	–	0.00 (0.00)	0.02 (0.19)	0.01 (0.33)	0.06 (0.00)
ORES	0.03	–	0.00 (0.27)	0.09 (0.41)	0.01 (0.00)
ISR	0.06	0.06	–	0.22 (0.01)	0.00 (0.18)
AUS	0.19	0.21	0.23	–	0.23 (0.30)
SWI	0.03	0.03	0.05	0.18	–


*Values above the diagonal are *Q*_ST_ in Toronit and Greina (in parenthesis) and below the diagonal are *G*_ST_ for RFLP markers.*

### Effects of Local Temperature on Aggressiveness Evolution in *Z. tritici*

**FIGURE 3:**
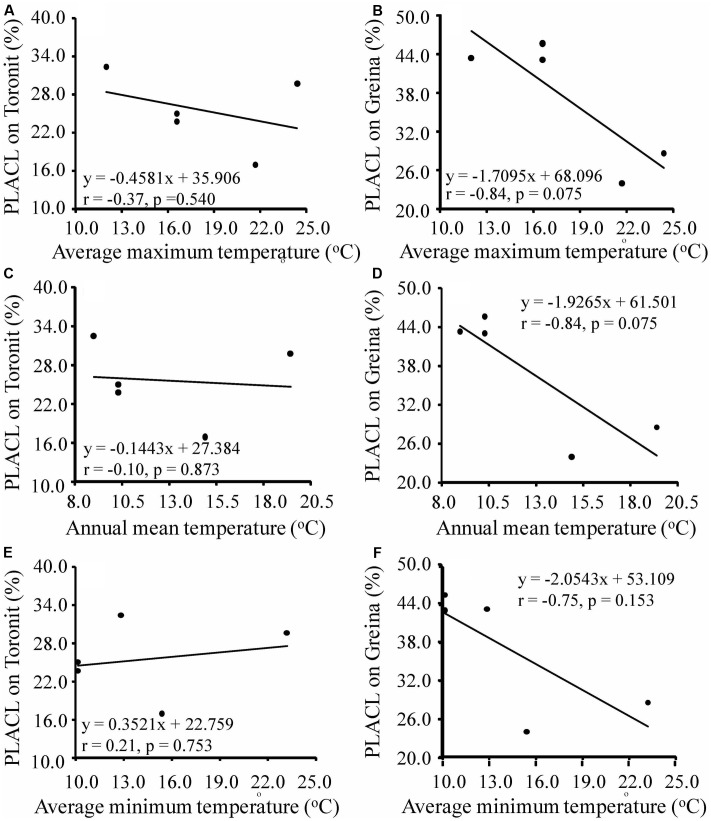
Correlation between maximum, mean and minimum annual temperatures at collection sites and mean aggressiveness measured by the percentage of leaf area covered by lesion of five *Z. tritici* populations on a moderately resistant cultivar Toronit **(A,C,E)** and a susceptible cultivar Greina **(B,D,F)**.

**FIGURE 4:**
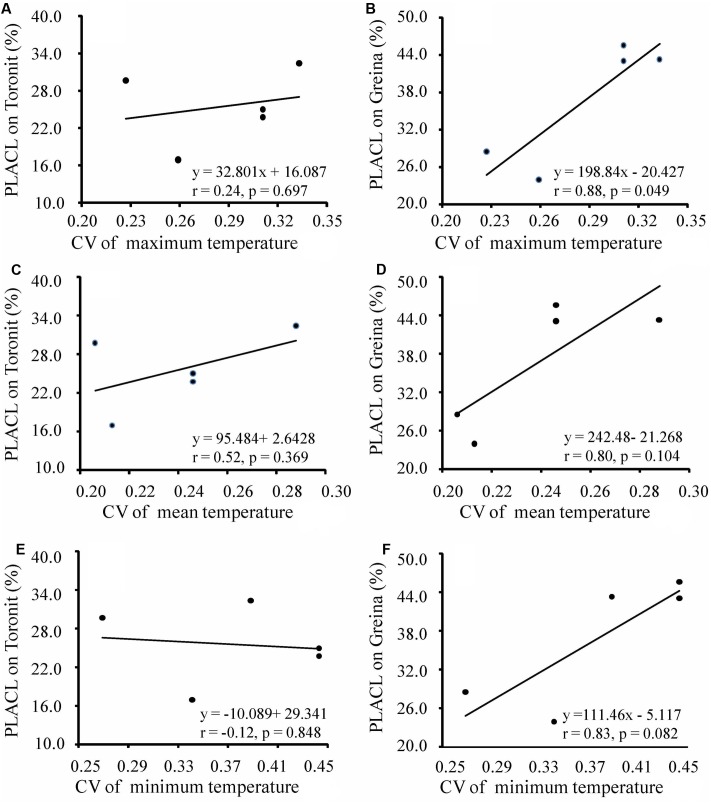
Correlation between coefficient of variance in temperatures at collection sites and mean aggressiveness measured by the percentage of leaf area covered by lesion of five *Z. tritici* populations on a moderately resistant wheat cultivar Toronit **(A,C,E)** and a susceptible wheat cultivar Greina **(B,D,F)**.

Annual mean, maximum and minimum temperatures in the five locations ranged from 9.0 to 14.9, 12.0 to 24.4 and 5.1 to 13.8 and coefficients of variation in the mean, maximum and minimum temperatures in the locations ranged from 0.206 to 0.288, 0.227 to 0.333 and 0.269 to 0.443 (Supplementary Table S1), respectively. Different patterns of temperature-aggressiveness associations were found in susceptible and resistant wheat cultivars. No associations were detected between aggressiveness and collection site temperatures on the moderate resistant cultivar (**Figures 3**, **4**). On the susceptible cultivar, however, pathogen mean aggressiveness was negatively correlated with collection site temperatures with a *p*-value ranging from 0.075 to 0.153 (**Figures 3B,D,F**) but positively correlated with the coefficient of variance in temperatures with a *p*-value ranging from 0.049 to 0.104 (**Figures 4B,D,F**).

## Discussion

The heritability of aggressiveness in the *Z. tritici* population originating from a moderately resistant cultivar in Oregon (ORER) was 13% [(0.633-0.562)/0.562] higher on the moderately resistant tester cultivar Toronit than that on the susceptible tester Greina (**Table 1**). Equally, the heritability of aggressiveness in the *Z. tritici* population originating from a susceptible cultivar in Oregon was 15% [(0.652-0.565)/0.565] higher on the susceptible tester cultivar Greina than that on the moderately resistant Toronit. This result suggests that *Z. tritici* has ability to adapt quickly to host resistance and that any new resistance genes deployed could be overcome by the pathogen in a short period of its commercial uses. Similar observations have been made in a number of other plant-pathosystems such as in grapevine-*Plasmopara viticola* interaction (Delmotte et al., 2014).

Different relationships between genetic differentiation in the RFLP marker and aggressiveness of *Z. tritici* populations were found on the moderately resistant cultivar Toronit and the susceptible cultivar Greina (**Figure 2**), supporting the hypothesis of host resistance-mediated evolution of pathogens documented also in other systems (Poulin et al., 2011; Delmotte et al., 2014). On the moderately resistant cultivar, *Q*_ST_ in aggressiveness was significantly lower than *G*_ST_ in RFLP (**Figure 2A**), suggesting a constraining selection which acts to spatially homogenize the pathogen’s population genetic structure. Previous studies showed that the moderately resistant cultivar displayed continuous variation in susceptibility to *Z. tritici* isolates (Zhan et al., 2016), suggesting the resistance is likely to be race non-specific as demonstrated in many plant quantitative resistance systems (Chartrain et al., 2004; Caffier et al., 2014). In race non-specific resistance, the host is expected to exert a similar level of selection on pathotypes derived from all geographic locations, leading to an overall lower *Q*_ST_ than *G*_ST_. Furthermore, selection by the moderately resistant cultivar is apparently weak as evidenced by a positive and significant correlation between pairwise *Q*_ST_ in aggressiveness and pairwise *G*_ST_ in RFLP (*r* = 0.71, *p* = 0.021; **Figure 2A**). Assuming other evolutionary forces are similar, the positive associations between *Q*_ST_ and *G*_ST_ are expected to get stronger as the levels of selection reduce. Theoretically, a perfect correlation (*r* = 1.00) between the two parameters should be observed when there is no selection on aggressiveness. Constraining and weak selection reduces the evolutionary speed of aggressiveness. However, quantitative resistance in hosts can erode due to constant evolution of pathogens and its durability in commercial productions also depends on ecological and epidemiological processes associated with particular host-pathogen interactions (Brown, 2015).

In contrary to the moderately resistant cultivar, *Q*_ST_ in aggressiveness was significantly higher than *G*_ST,_ in the RFLP marker on the susceptible cultivar and no correlation was detected between *Q*_ST_ and *G*_ST_ (**Figure 2B**), indicating directional and stronger selection for local adaptation (Yang et al., 1996; Zhan et al., 2005) by the susceptible cultivar. These patterns of natural selection in aggressiveness on the susceptible cultivar were unexpected. Theoretically all pathogen pathotypes are able to infect susceptible host genotypes, leading to weak host selection. However, stronger host selection imposed by susceptible cultivars rather than moderately resistant cultivars has also been documented previously both in wheat-*Z. tritici* (Ahmed et al., 1996; Zhan et al., 2002) and other plant-pathogen systems (Abang et al., 2006), suggesting it could be a common phenomenon in host-pathogen interactions. For example, in a mark-release-recapture field experiment involving the wheat-*Phaeosphaeria nodorum* system, it was found that pathogen populations recovered from susceptible cultivars showed higher selection coefficients distributed in a wider range and quicker change in genotype frequency than those recovered from moderately resistant cultivars within the same 2-year period (Sommerhalder et al., 2011). It has been reported that many susceptible cultivars contain ‘defeated’ qualitative resistance (Lozoya-Saldana, 2011) and stronger selection by susceptible host genotypes may result from the residual effects of the defeated qualitative resistances (Brodny et al., 1986; Pedersen and Leath, 1988).

The hypothesis of host resistance-mediated evolution is also supported by the analysis showing a different pattern of associations between genetic variation and aggressiveness of the pathogen populations on the moderately resistant and susceptible cultivars. Aggressiveness of *Z. tritici* was positively correlated to its genetic variation on the moderately resistant cultivar but no such association was found on the susceptible cultivar (**Figure 1**). Genetic variation in life history traits constitutes the basis for the adaptive potential of species (McGuigan, 2006; Boldogkoi, 2007). Fisher’s fundamental theorem of natural selection states that the adaptability of a species to changing environments depends on its additive genetic variance in ecological and morphological characters that are relevant to fitness (Fisher, 1958). A positive correlation between mean aggressiveness and genetic variation of *Z. tritici* on the moderately resistant cultivar suggests that pathogen populations with high genetic variation perform better in overcoming host defense systems and inducing significant levels of disease. Although rarely studied in pathogens, positive associations between genetic variation and the mean performance of populations have been documented in several host systems (Tiira et al., 2003; Vandewoestijne et al., 2008; Blomquist, 2009). For example, decreased genetic diversity in butterfly populations resulted a sharp reduction in adult lifetime expectancy (Vandewoestijne et al., 2008), a key component of individual fitness. In plant-pathogen interactions, increasing genetic variation of hosts by cultivar mixture reduced disease development (Zhan and McDonald, 2013) and increased seed production (Zhu et al., 2000). Inbreeding depression is a common phenomenon in nature (Rfa, 1986; Astete and De, 2002) and high genetic variation in populations reduces the chance of genetically related individuals to mate. Positive association between population performance and genetic variation in the current and previous studies may results from the reduced inbreeding rate in populations with high variation.

We also found that local temperatures interact with host resistance together in determining the evolution of aggressiveness in wheat-*Z. tritici* system, consisting with results seen in other host-pathogen interactions (Harvell et al., 2002; Mboup et al., 2012; Schade et al., 2014). The pathogen aggressiveness on the partial resistant cultivar, Toronit, was always total unrelated to any measures of temperatures at the collection sites including maximum, mean and minimum temperatures (**Figures 3A,C,E**, **4A,C,E**). In contrast, on the susceptible cultivar Greina, the *p*-values between aggressiveness and local temperatures ranged from 0.075 to 0.153 (**Figures 3B,D,F**) and between the coefficient of variance in maximum, mean and minimum temperatures at collection sites and mean aggressiveness ranged from 0.049 to 0.104 (**Figures 4B,D,F**). In statistics, though 0.05 level is adopted in most studies, other levels (e.g., 0.1 and 0.01) are also used (Perezgonzalez, 2015). In the ecological and evolutionary studies which are usually constrained by small sample size, 0.1 level has been often used (Johnson et al., 2009; Walker et al., 2014). Only five data points were included in the current study and we consider most of the correlations between pathogen aggressiveness on the susceptible cultivar and local temperatures are significant but further studies involved more data points (populations) are required to confirm the result. The finding that local temperatures at the collection sites do not affect the aggressiveness development of *Z. tritici* on partially resistant cultivar reinforces that quantitative resistance can slow down the evolution of pathogen.

The finding of negative association between aggressiveness and temperature was unexpected and suggests a counter-gradient adaptation of the pathogen to temperature as a result of a trade-off among pathogen aggressiveness, energy relocation and seasonality. Such trade-offs have also been found in other species such as the Irish famine pathogen *Phytophthora infestans* (Yang et al., 2016) and *Fusarium pseudograminearum* (Sabburg et al., 2015). It has been widely suggested that increasing air temperatures due to anthropogenic activity may significantly raise the occurrence, severity and epidemics of plant diseases (Mboup et al., 2012; Gautam et al., 2013). Negative associations of aggressiveness (**Figure 3**; Sabburg et al., 2015), pathotype complexity (Wu et al., 2016), infectivity frequency (Wu et al., 2016) and pesticide tolerance (Qin et al., 2016) with local temperatures raise the possibility that increases in air temperature during global warming may have a negative effect on the evolution of pathogens. Whether this is a universal phenomenon or it is only specific to the few systems studied is worthy of further investigation.

## Author Contributions

FC analyzed the data and wrote the manuscript; G-HD and D-LL wrote the manuscript; and JZ conceived and designed the experiments, analyzed the data and wrote the manuscript. All authors reviewed the manuscript.

## Conflict of Interest Statement

The authors declare that the research was conducted in the absence of any commercial or financial relationships that could be construed as a potential conflict of interest.

## Footnotes

1 http://www.worldclimate.com/
