# Supplementary material for: Host Resistance and Temperature-Dependent Evolution of Aggressiveness in the Plant Pathogen Zymoseptoria tritici
Source: Front Microbiol. 2017 Jun 28;8:1217. doi: 10.3389/fmicb.2017.01217 (PMC5487519; doi:10.3389/fmicb.2017.01217)
Supplement: Supplementary file 1 [file Table_1.DOCX]

Supplementary material

Table S1 Annual temperatures (maximum, mean and minimum) and their coefficient of variation at collection sites

| Population | Collection site | Annual temperature  (^o^C) | | |  | Coefficient of variation | | |
| --- | --- | --- | --- | --- | --- | --- | --- | --- |
|  |  | Max. | Mean | Min. |  | Max. | Mean | Min. |
| AUS | Wagga Wagga | 21.7 | 14.9 | 8.6 |  | 0.259 | 0.213 | 0.341 |
| ISR | Nahal Oz* | 24.4 | 19.4 | 13.8 |  | 0.227 | 0.206 | 0.269 |
| ORER | Corvallis, Oregon | 16.6 | 10.3 | 5.1 |  | 0.311 | 0.246 | 0.443 |
| ORES | Corvallis, Oregon | 16.6 | 10.3 | 5.1 |  | 0.311 | 0.246 | 0.443 |
| SWI | Berga Irchel, Winterthur | 12.0 | 9.0 | 6.9 |  | 0.333 | 0.288 | 0.388 |

* Temperature data for Beer-Sheva, a location approximately 50 km east of Nahal Oz was used because of no available information for Nahal Oz. Max. and Min. represents maximum and minimum temperature, respectively.
